# A 2D nanoflower-like ordered mesoporous Bi_12_ZnO_20_ catalyst with excellent photocatalytic antibacterial properties

**DOI:** 10.1128/spectrum.00625-24

**Published:** 2024-07-09

**Authors:** Jingmei Li, Shuai Liu, Chenming Zhan

**Affiliations:** 1 Changchun University of Science and Technology, Changchun, China; Ocean University of China, Qingdao, China

**Keywords:** photocatalysis, Bi_12_ZnO_20_, antibacterial, nanoflower

## Abstract

The ordered mesoporous ZnO was successfully synthesized using the template method in this article, and Bi ions were etched into ZnO to form two-dimensional nanoflower structures of Bi_12_ZnO_20_ with NA_3_SSA as a guiding agent. The crystal structure, morphology, and optical properties of the photocatalyst were characterized by X-ray diffractometer (XRD), scanning electron microscope (SEM), energy-dispersive spectrometer(EDS), and ultraviolet-visible diffuse reflectance spectrum (UV-vis DRS). Under illumination conditions, the obtained materials exhibited excellent bactericidal ability against both gram-positive and gram-negative bacteria, as well as effective inhibition against fungi. Among them, the bactericidal effect of *Pseudomonas aeruginosa* was found to be the most rapid, achieving a sterilization rate of 100% within 30 min of light irradiation. Even after three cycles of antibacterial activity testing, the Bi_12_ZnO_20_ material still demonstrated good photocatalytic performance. The nanoflower-shaped materials provide an enhanced fluid adsorption capacity and more active centers for photocatalytic reactions while also improving light absorption capacity, photogenerated electron-hole separation efficiency, and electron transport efficiency. The cytotoxicity assessment of Bi_12_ZnO_20_ revealed no significant toxic effects. Therefore, this study presents a nanoflower-shaped material with highly efficient photocatalytic antibacterial properties for applications in production and daily life; it holds significant importance in eliminating harmful bacteria and plays a crucial role in environmental protection.

**IMPORTANCE:**

The flower-shaped photocatalytic material Bi_12_ZnO_20_, consisting of nanoparticles, was successfully synthesized in this study. Rigorous antibacterial experiments were conducted on various fungi using the material, yielding excellent results. Furthermore, the application of this material for antibacterial treatment of livestock and poultry manure sewage in real-life scenarios demonstrated remarkable efficacy.

## INTRODUCTION

Photocatalysis technology emerged in the 1970s when Fujishima fortuitously discovered that irradiating a titanium dioxide (TiO_2_) electrode with ultraviolet light could effectively decompose water. This groundbreaking discovery not only propelled TiO_2_ to the forefront of photocatalysis research but also popularized this environmentally friendly green technology for environmental protection (1). In subsequent decades, researchers have extensively investigated photocatalysis technology by exploring diverse semiconductor materials and distinct principles underlying photocatalytic processes. As a result, they have enhanced its efficiency and achieved remarkable advancements across various application domains such as hydrogen production through water decomposition, carbon dioxide reduction, pollutant degradation in water, and antibacterial photocatalysis (2, 3).

Metal oxides, particularly semiconductor metal oxides such as TiO_2_ and ZnO, are widely utilized as catalysts with excellent photocatalytic oxidation performance (4). Among them, ZnO stands out due to its remarkable advantages including large storage capacity, great stability, and high catalytic activity (5). As a result, it finds extensive applications in various fields such as wastewater treatment, antibacterial processes, and self-cleaning technologies (6, 7). However, the utilization of ZnO is limited by its reliance on ultraviolet irradiation for valence band electron transition and subsequent enhancement of photocatalytic performance (8, 9). Unfortunately, the quantum efficiency of ZnO remains relatively low, which hampers its practical application and limits its photocatalytic potential under sunlight exposure (10, 11).

ZnO, the primary zinc oxide compound, exhibits three distinct crystal structures: cubic rock salt structure (rock salt), cubic sphalerite structure (Zincblende), and hexagonal wurtzite structure (12). The cubic rock salt structure (rock salt) is a rare crystal arrangement that remains stable solely under high-pressure conditions (13). Conversely, the hexagonal Wurtzlt structure is the most prevalent crystal configuration in ZnO due to its densely packed atoms and inherent stability (14, 15). However, ZnO possesses a wide band gap ranging from 3.05 to 3.37 eV, resulting in photogenerated electrons being produced only under ultraviolet irradiation for achieving antibacterial purposes (16). Consequently, Bi^3+^-doped ZnO emerges as an innovative strategy for developing efficient Bi-based photocatalysts while also enabling control over the morphology of Bi_12_ZnO_20_ materials (17). This catalyst holds significant potential for application in combating harmful bacteria (18, 19).

Environmental pollution has climbed to the top of the global priority list in recent years, and disease-causing microorganisms are contaminating water supplies at an alarming rate, a worrying situation for humans and all other organisms that depend directly or indirectly on water (5, 20). Because photocatalysis technology is cost-effective and ecologically acceptable, it has also received a lot of attention in the field of removing disease-causing microorganisms from water environments (21, 22). Zhang et al. proposed an integrated antibacterial nanotechnology without pollution residues, which synergistically enhances the antibacterial activity of ceftazidime by using the inorganic nano-Ag3PO4, in 2019, and subsequently removes drug residues by photocatalysis. (23). In 2021,(24) . prepared Fe_3_O_4_@PDA@Ni-DT composite material (24). Fe_3_O_4_@PDA@Ni-DT composite had a killing rate of 99.3% against gram-negative *Escherichia coli* and 98.8% against gram-positive *Staphylococcus aureus* (25, 26). Due to the introduction of nano-Fe_3_O_4_, the composite material maintained excellent recyclability (4, 27, 28). With its superior stability, strong oxidation ability, and high catalytic activity, photocatalytic materials have shown broad prospects for applications in antibacterial treatment, cleaning processes, air purification, and other fields (23, 29).

## MATERIALS AND METHODS

Reagents from Xinhua Research Institute, China, were utilized in the experiments. Ethanol (A.R., Beijing Chemical Works), zinc acetate dehydrate (A.R., Longxi Chemical Reagent Co., Ltd., China), lithium hydroxide (A.R., Tianjin Yaohua Chemical Plant, China), sodium hydroxide (A.R., TianJin GuangFu Technology Development Co., Ltd., China), sodium sulfosalicylate (Na_3_SSA, A.R., Xilong Chemical Reagent Co., Ltd., China), and bismuth nitrate pentahydrate [Bi(NO_3_)_3_·5H_2_O, A.R., Xiqiao Science Co., Ltd.] were employed as reagents in the experiments, whereas deionized water was used as a solvent.

### Characterization

Ordered mesoporous ZnO: add 1.0 g of cetyltrimethylammonium bromide (CTAB) to 480 mL of deionized water and stir at a temperature of 80°C until the solution achieves uniformity. Subsequently, dissolve 3.5 mL of sodium hydroxide (NaOH) with a concentration of 2 mol/L in the solution, followed by gradual addition of zinc acetate dihydrate (0.02 mol) while stirring for 1 h. Then, a certain amount of lithium hydroxide (LiOH) is introduced into the mixture. After adjusting the pH value to 10 using ordered mesopore node solution (with pH values tested at intervals including 9, 10, 11), continue the reaction for an additional hour before filtering, washing, and drying the resulting products in an oven set at a temperature of 60°C. Finally, transfer the dried products into a crucible and place them in a Muffle furnace where they will be heated gradually at a rate of 1°C/min until reaching a calcination temperature of 550°C for 4 h to obtain the precursor material for ordered mesoporous ZnO.

**Fig 1:**
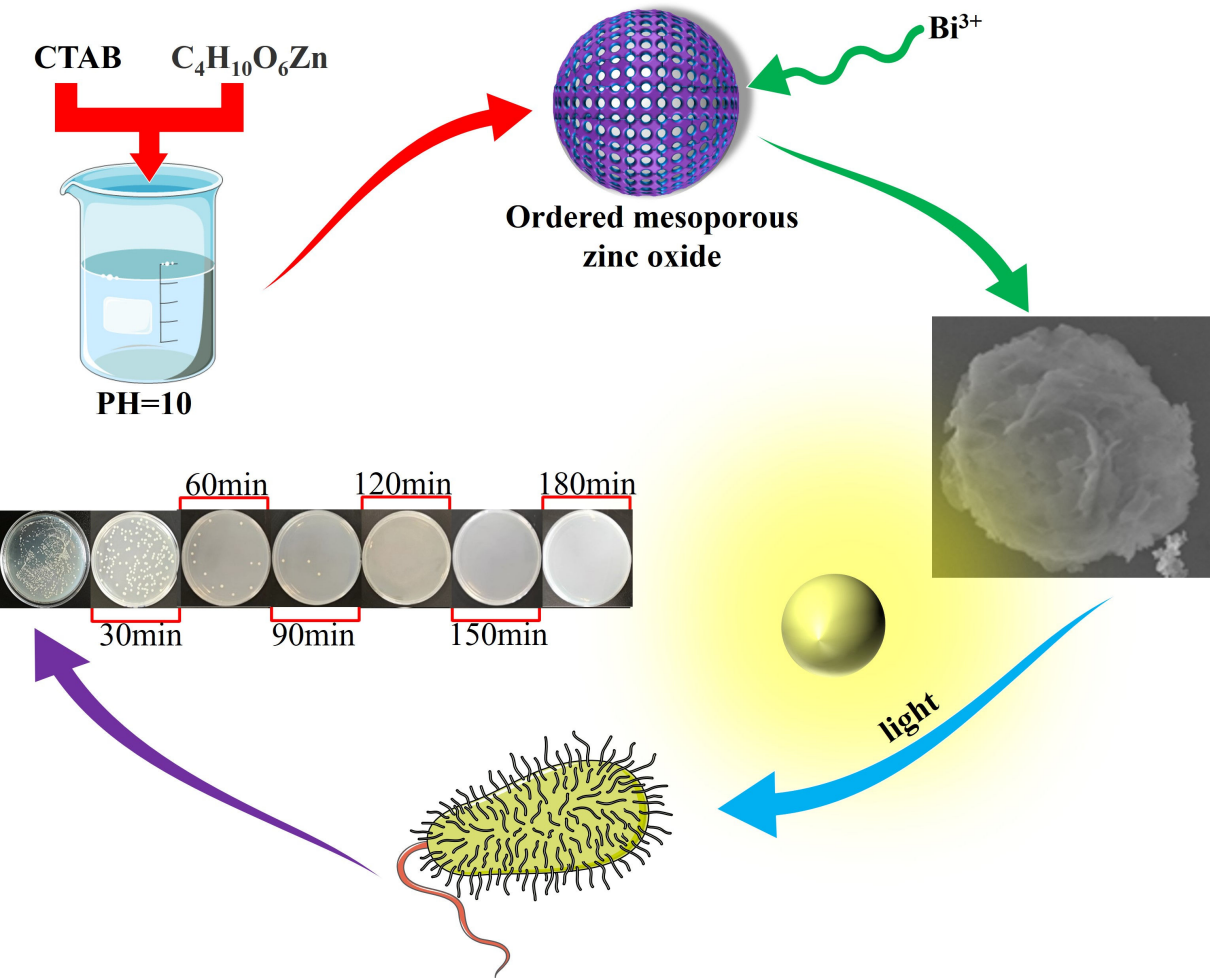
Experimental flow diagram.

Synthesis of Bi_12_ZnO_20_ (17): the prepared ordered mesoporous ZnO precursor, with a quantity of 0.01 mol, was dispersed into deionized water using ultrasound to ensure a uniform dispersion. Subsequently, it was dissolved by adding varying proportions of Bi(NO_3_)_3_·5H_2_O and sodium sulfosalicylate, followed by continuous stirring for 24 h. The resulting mixed solution was then transferred to a stainless steel elevated-pressure reactor lined with polytetrafluoroethylene and subjected to hydrothermal reaction at different temperatures for specific durations. The resultant white powder was thoroughly washed and filtered multiple times using deionized water and anhydrous ethanol before being dried in an oven at 60°C. To enhance the crystallinity of the material and eliminate any organic residue, the dried product underwent calcination in a crucible placed inside a Muffle furnace set at 220°C for 12 h, followed by further calcination at 500°C for another 4 h until the desired sample was obtained. The specific experimental process is shown in Fig. 1. The diagram elements are from the experiment.

### Structural characterization

The crystal structure of the sample was analyzed using a Rigaku Smart-Lab type X-ray diffractometer (XRD) (30). The morphology, structure, and elemental composition distribution of the composites were observed using a Hitachi Regulus 8100 scanning electron microscope (SEM) and Bruker XFlash 6l60 X-ray energy-dispersive spectrometer (EDS). The light response range of Cary 500 ultraviolet-visible diffuse reflectance spectrum (UV-vis DRS), prepared by Varian Company in the USA, was examined. The microstructure of the prepared samples was characterized using transmission electron microscopy (TEM) with a JEM·2010 TEM model. Elemental composition, valence states, and chemical bonds on the surface of the sample were analyzed using X-ray photoelectron spectroscopy (XPS) with an ESCALAB 250 model and Al Ralph as the X-ray source.

### Photocatalytic antibacterial experiment

The antibacterial properties of the materials were determined using the gradient dilution method and plate colony counting method. The cultured bacterial solution was diluted stepwise by factors of 10^2, 10^3, 10^4, 10^5, 10^6, 10^7, 10^8, and 10^9 in lysogeny broth (LB) liquid medium solution (31). Each dilution (100 µL) was evenly coated on solid medium and was incubated for 18 h to allow individual colonies to form. Promptly documenting and monitoring the number of bacterial communities are imperative. Based on the final results, a 10-fold dilution factor of 6 was chosen for designing the experiment.

The photocatalytic antibacterial activity of the composite against *E. coli*, *S. aureus*, *Bacillus subtilis*, *Pseudomonas aeruginosa*, and *Candida albicans* was assessed using the plate colony counting method. *E. coli* and *S. aureus* represent typical gram-negative and gram-positive bacteria, respectively. *B. subtilis* is a common soil bacterium found in decomposing organic matter, whereas *P. aeruginosa* is often associated with metal surfaces in marine environments. *C. albicans* is a dimorphic fungus.

LB medium was used as the bacterial nutrient source, whereas Martin medium served as the fungal nutrient source. All experiments were conducted in triplicate simultaneously. Prior to experimentation, all glassware and equipment underwent sterilization at 121°C for 20 min. The procedure of the photocatalytic antibacterial experiment involved weighing and adding the samples to a 50-mL beaker, followed by addition of 15 mL of 0.9% NaCl solution to ensure a uniform dispersion through ultrasound treatment for 15 min. Subsequently, a volume of 5 mL bacterial solution was extracted from the cultured bacterial solution and was added into the sample-containing beaker to facilitate complete contact between bacteria and samples. A fixed-speed stirring was maintained throughout the experiment while utilizing a vertically positioned light-emitting diode (LED) lamp with an intensity of 35 W as the light source. At predetermined time intervals, a 1-mL volume of mixture was withdrawn and diluted with liquid medium at a ratio of 10^6 times. The resulting solution was thoroughly shaken and uniformly coated onto plates using 100-µL aliquots before being placed upside down in an incubator set at a constant temperature for cultivation purposes. The bacterial culture conditions were as follows: temperature maintained at 37°C for a duration of 18 h; fungal culture conditions involved incubation at 28°C for 48 h. A control group was established in this experiment, consisting of 15 mL of sterile saline solution (0.9% NaCl) without any samples, along with the addition of a bacterial solution (5 mL). Time sampling was also conducted at the beginning of light exposure. Finally, by analyzing changes in colony numbers during different time points under illumination, the antibacterial efficiency of photocatalysis could be calculated accurately.See equation that follows:

Antibacterial efficiency = (number of colonies without adding sample light – number of colonies after adding sample light) / number of colonies without adding sample light × 100% (32).

### MTT cytotoxicity assay

Normal 3T3 skin fibroblasts were carefully selected for analysis. The experiments were conducted using 96-well plates, with a cell density of approximately 5,000 cells per well and a volume of 100 µL per well. The cells were incubated under controlled conditions at 5% CO_2_ and were maintained at a temperature of 37°C until they adhered to the plate surface after a period of 12 h. Subsequently, a solution containing the desired material was added to each well (100 µL) and cultured for an additional duration of 24 h. Finally, the optical density (OD) values at a wavelength OD of 490 nm were measured to assess cellular responses in each individual well.

### Treatment of livestock and poultry excrement wastewater

The practical antibacterial performance of the material was assessed by using livestock and poultry sewage as the authentic water sample to evaluate its efficacy in removing and eradicating pathogenic microorganisms. Specifically, the material was introduced into diluted livestock and poultry sewage from a farm, followed by LED light treatment. Subsequently, 100 µL of treated water was extracted and spread onto solid LB plates for colony counting analysis to determine its antibacterial effectiveness.

### Experiment on natural light

To evaluate the efficacy of the chemical material under real sunlight conditions, *E. coli* was used as a model bacterium to assess its antibacterial ability of photocatalysis when exposed to direct solar irradiation (25). The material was added to the *E. coli* culture medium for conducting the aforementioned antibacterial experimental procedures.

## RESULTS

### Optimal synthesis ratio

**Fig 2:**
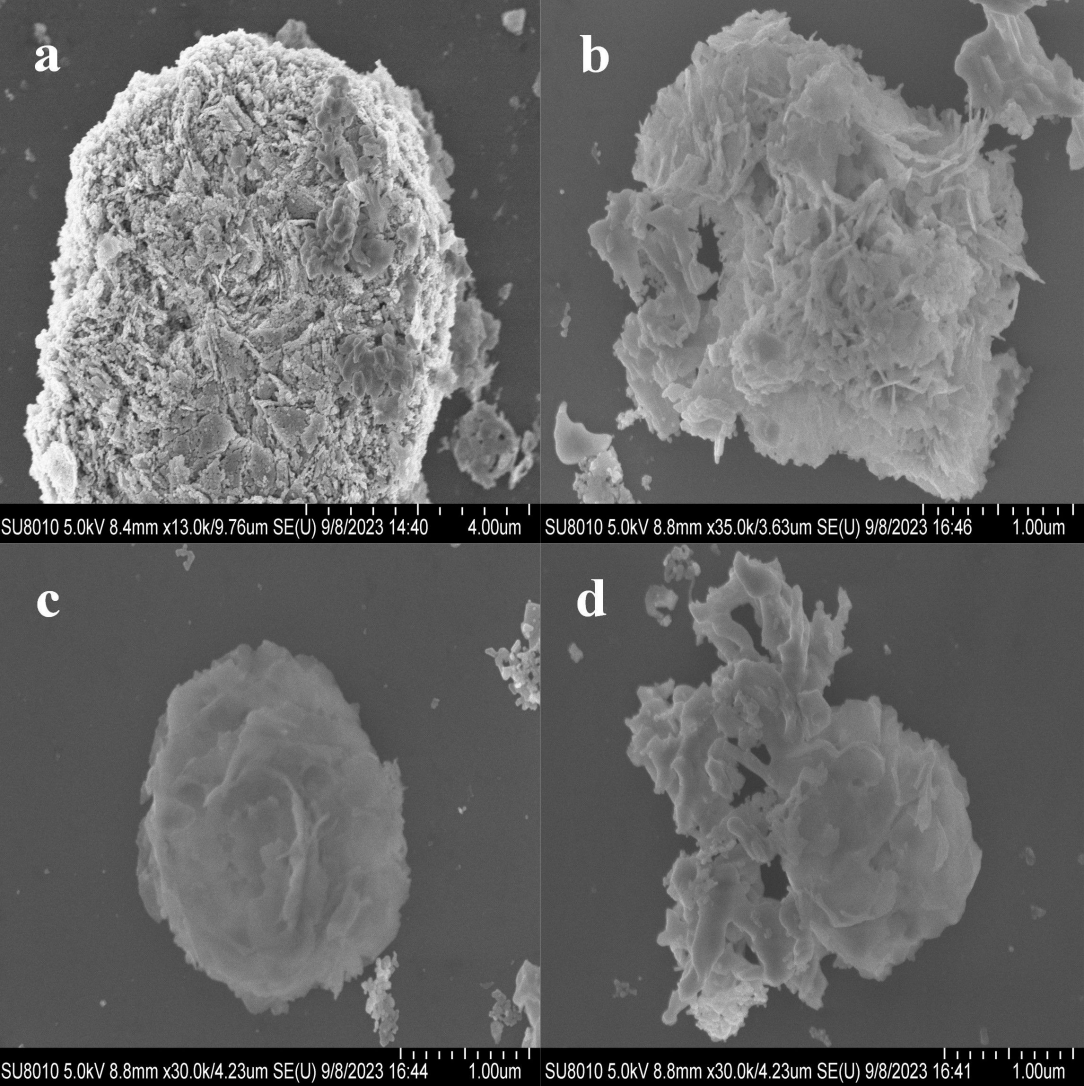
SEM image of the produced sample (**a**) NA_3_SSA-ZnO. Zn:Bi molar ratios of (b) 1:0.2, (c) 1:0.3, and (d) 1:0.4.

Based on the analysis of SEM scans and electrochemical test results, the synthesized sample was evaluated to determine the optimal preparation’s cumulative proportion (33). The morphology of the prepared material was examined using a scanning electron microscope, as depicted in Fig. 2. Figure 2a displays the SEM image of NA_3_SSA-ZnO, revealing a significant etching effect that results in the formation of numerous pores within the mesoporous ZnO structure. In Fig. 2b through d, the SEM images depict the Zn:Bi molar ratios of 1:0.2, 1:0.3, and 1:0.4, respectively, clearly demonstrating abundant Bi ion nanosheets onto the mesoporous ZnO framework across all material proportions. Notably, at a ratio of 1:0.3, an enhanced arrangement of nanosheets integrated with mesoporous ZnO is observed, effectively enveloping the entire surface area and forming a distinctive nanoflower-like structure. This architectural design augments photoadsorption sites on the material’s surface while concurrently enhancing photocatalytic activity. The synthesized nanoflower-like structures possess an approximate diameter of 2 μm with individual nanosheets measuring around 50 nm in size; their surfaces exhibit relative smoothness, thereby providing an optimal reaction environment for efficient photocatalytic reactions. Fig. 2c has been used in Fig. 1 schematic for convenience of expression.

**Fig 3:**
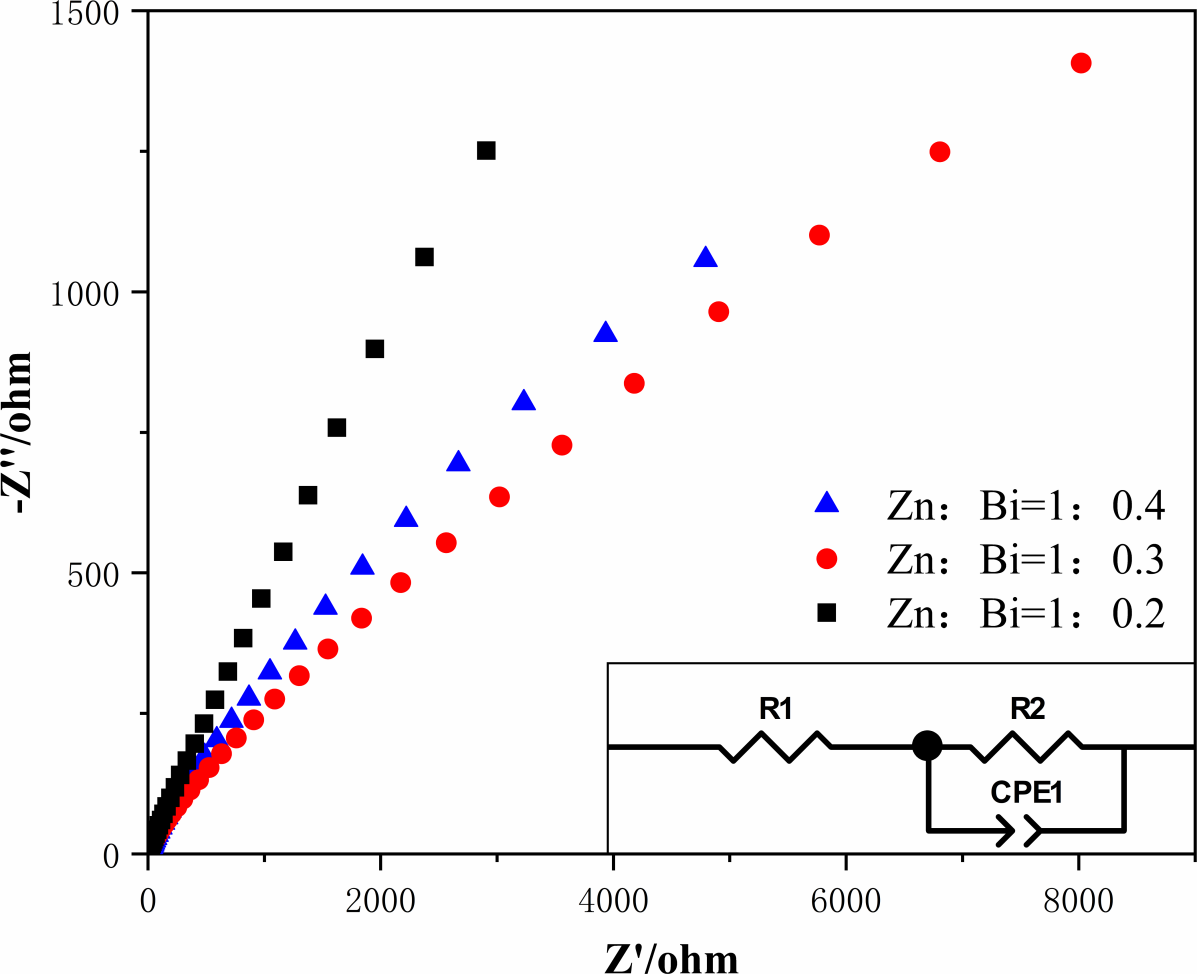
Electrochemical impedance diagram of three proportional synthetic materials.

Electrochemical impedance spectroscopy (EIS) tests revealed the photogenerated charge migration ability of the prepared sample at the solid/electrolyte interface. A smaller arc radius in the impedance spectrum indicates enhanced electron-hole separation and improved photocatalytic performance. As depicted in Fig. 3, the arc radius diameter for a Zn:Bi molar ratio of 1:0.3 is smaller compared to the other two ratios, suggesting that materials with this composition exhibit a more efficient photogenerated charge separation and faster interfacial charge transfer rates.

### Structural characterization of the catalyst

**Fig 4:**
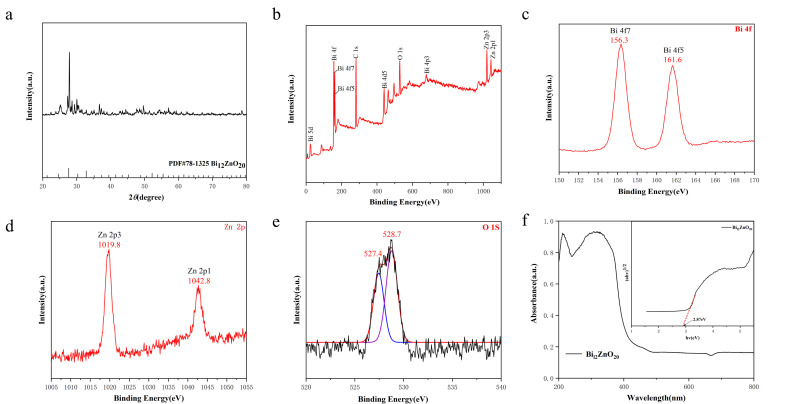
(**a**) XRD diffraction patterns of the samples. (**b**) XPS full spectrum and XPS spectra of (**c**) Bi-4f state, (**d**) Zn-2p state, and (**e**) O1s state of sample. (**f**) Solid UV and forbidden bandwidth of the sample.

To analyze the crystal structure of the prepared samples, XRD tests were performed (34). As depicted in Fig. 4a, the diffraction peaks observed at 2θ = 21.30°, 24.65°, 27.61°, 30.31°, 32.81°, 39.45°, 41.46°, 43.40°, 45.27°, 48.84°, 53.85°, and 55.45° correspond to the crystal plane diffractions of Bi_12_ZnO_20_: (211), (220), (310), (222), (321), (024), (332), (422), (510), (521), (442), and (532). These findings are consistent with the characteristic diffraction pattern of cubic phase Bi_12_ZnO_20_ as reported in literature reference PDF#78-1325.

To further determine the elemental composition and valence state of Bi_12_ZnO_20_, XPS was employed in this study to characterize the sample. The resulting test data are presented in Fig. 4, which displays signal peaks for Bi, Zn, and O elements in the total spectrum Fig. 4b, indicating that the prepared material consists of these three elements. Additionally, high-resolution XPS spectra for Bi 4f, Zn 2p, and O 1s are shown in Fig. 4c through e, respectively. As depicted in Fig. 4c, the characteristic peaks observed at binding energies of 156.3 and 161.6 eV can be attributed to the energy level characteristics of Bi’s 4f7 and 4f5, respectively. Figure 4d displays two distinct characteristic peaks: one at a binding energy of 1,019.8 eV corresponding to Zn’s 2p3, and another at a binding energy of 1,042.8 eV representing Zn’s 2p1; this indicates that Zn exists in its elemental state within the photocatalyst. In Fig. 4e, it is evident that the characteristic peaks located at binding energies of 527.4 and 528.7 eV belong to O’s energy level characteristics for its respective O1s peak; specifically, the O1s peak at a binding energy of 527.4 eV can be attributed to Bi–O–Bi interactions, whereas the O1s peak at a binding energy of 528.7 eV is associated with adsorbed oxygen species such as OH^-^ or O on the surface of the Bi_12_ZnO_20_ compound.

To evaluate the photocatalytic performance, we employed UV-vis DRS analysis to determine the band gap width of the material. Figure 4f illustrates the UV-vis DRS spectrum of the sample, revealing its predominant light absorption below 490 nm and thus indicating a response to visible light. The band gap energy (Eg) was estimated using αhv = A(hv–Eg)^n/2^ formula, where α represents the absorption coefficient, v denotes the frequency of light, Eg signifies the band gap energy, and A stands for the proportionality constant; n is determined by optical transition types in semiconductors (35). The application of this equation allows us to conclude that Bi_12_ZnO_20_ possesses a band gap width measuring at 2.87 eV.

**Fig 5:**
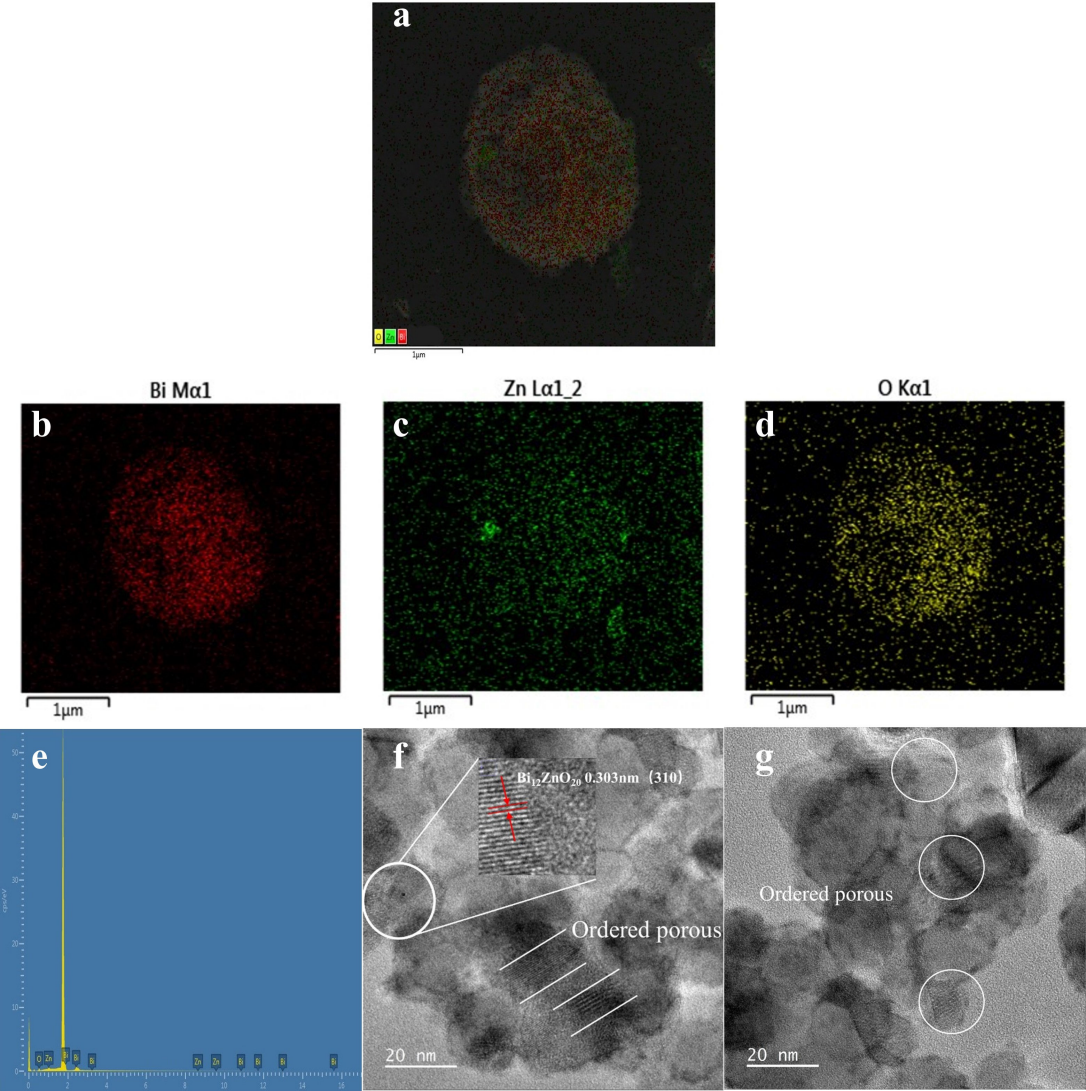
(**a**) SEM image, (**b–e**) images of distribution of elements, and (**f and g**) TEM images.

EDS was employed to analyze the presence of Bi^3+^ doping in the material and the elemental composition. Figure 5a through f illustrates the distribution of all elements (Bi, Zn, and O) within the Bi_12_ZnO_20_ material. Notably, Bi accounted for 13.03% of the total composition, signifying a successful synthesis of Bi_12_ZnO_20_ and an increased number of adsorption sites on its surface. This incorporation also introduced desirable properties associated with Bi^3+^, consequently enhancing the photocatalytic activity of this material.

In order to further investigate the structural characteristics of the photocatalyst prepared by our research institute, we conducted a high-resolution transmission electron microscopy (HRTEM) characterization on the samples. The test results shown in Fig. 5f and g clearly reveal an ordered arrangement of porous structures. In Fig. 5f, lattice fringes with a spacing of 0.303 nm can be observed, corresponding to the Bi_12_ZnO_20_ material’s (310) crystal plane as consistent with XRD results.

### Antibacterial effect and antibacterial mechanism of catalyst

**Fig 6:**
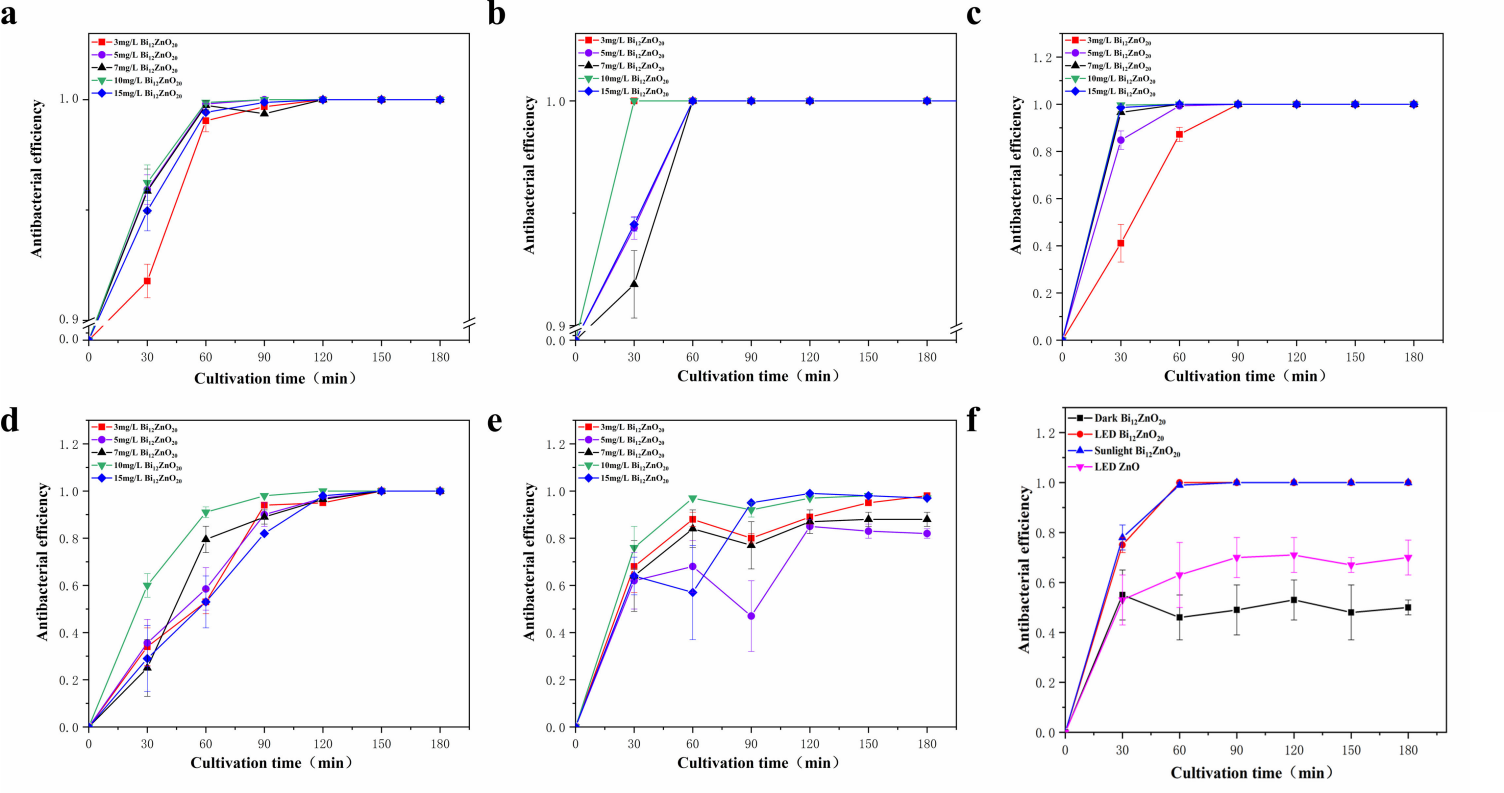
(a–e) Antibacterial efficiency of materials under LED light source. (f) Antimicrobial properties of materials under diverse light sources.

Based on the excellent photocatalytic activity exhibited by the synthesized materials, we conducted separate tests using *E. coli*, *S. aureus*, *P. aeruginosa*, *B. subtilis*, and *C. albicans* as target microorganisms. The antibacterial efficacy of Bi_12_ZnO_20_ under LED light is depicted in Fig. 6a through e.

From Fig. 6a, it can be inferred that *E. coli* can be completely eradicated within 1 h under the optimal antibacterial concentration of 1,000 mg/L, with a remarkable node sterilization rate of up to 96% in just half an hour. The outcomes depicted in Fig. 6b and c for *S. aureus* and *P. aeruginosa* at the same concentration demonstrate a sterilization rate of 100% within 30 min. Figure 6d demonstrates that the optimal concentration group exhibited a remarkable ability to eradicate *B. subtilis* within 2 h and subsequently achieved an equilibrium state. However, *C. albicans* material cannot be fully eradicated; only groups treated with concentrations of 1,000 and 1,500 mg/L achieved an approximately 98% elimination and tended toward stability, as illustrated in Fig. 6e. To investigate disparities between natural light and LED light sources in our laboratory setting, we conducted a comparative experiment. As shown in Fig. 6f, natural light proves more effective than LED light source, thereby highlighting the excellent photocatalytic antibacterial properties exhibited by this material.

**Fig 7:**
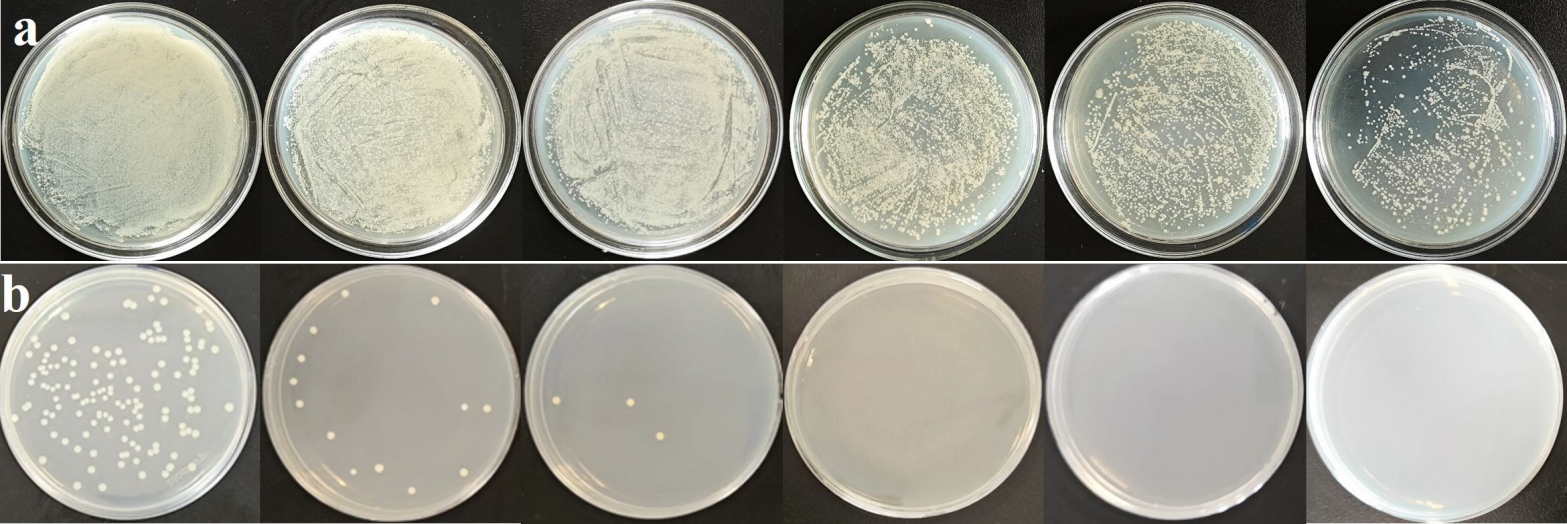
(**a**) The number of colonies after dilution of fecal water; from left to right, the dilution is 10, 100, 200, 400, 800, and 1,600 times. (**b**) Photocatalytic antibacterial effect of the material on diluted fecal water, from left to right, is 30, 60, 90, 120, 150, and 180 min.

Then, we proceeded to apply the aforementioned material to treating actual domestic sewage, specifically livestock wastewater. Initially, we determine the dilution factors of the bacterial solution using a solid agar plate method. As depicted in Fig. 7a from left to right, sequential dilutions were performed at ratios of 10-, 100-, 200-, 400-, 800-, and 1,600-fold. As depicted in Fig. 7b, the sterilization process of the sewage was observed with a material concentration of 1,000 mg/L at regular intervals of 30 min from left to right (36). Figure 7 has been used in Fig. 1 schematic for convenience of expression.

**Fig 8:**
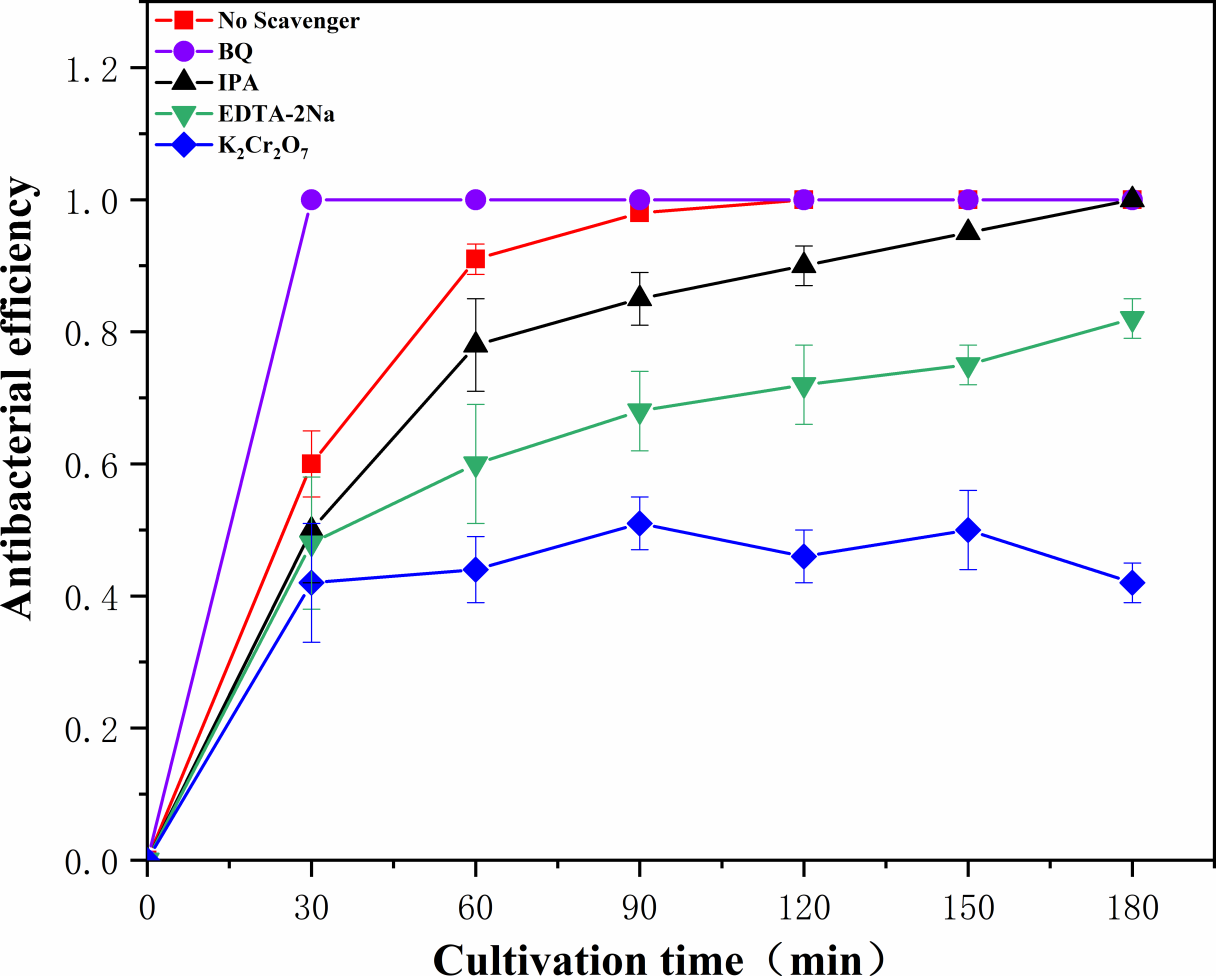
Free-radical capture experiments.

The photocatalytic degradation mechanism of materials was investigated through active species capture experiments. Figure 8 illustrates the quenching of ·OH, h^+^, ·O^2-^, and e^-^ using isopropyl alcohol (IPA), disodium ethylenediamine tetraacetate (EDTA-2Na), p-benzoquinone (BQ), and potassium dichromate (K_2_Cr_2_O_4_) as scavengers, respectively (37). The addition of K_2_Cr_2_O_4_ resulted in a decrease in antibacterial efficiency, indicating the significant role played by E-material in the photocatalytic antibacterial process (38). Conversely, EDTA-2Na and IPA had only a weak impact on antibacterial efficiency after their addition, suggesting that h^+^ and ·OH have minimal involvement in the photocatalytic antibacterial reaction. However, BQ is a broad-spectrum antibacterial agent that can interact with DNA and proteins inside cells, generating reactive oxygen species, which lead to protein denaturation and bacterial death. Additionally, the oxidative action of BQ can also cause mitochondrial damage, resulting in interference with the discussed outcomes.

### Cytotoxicity test

**Fig 9:**
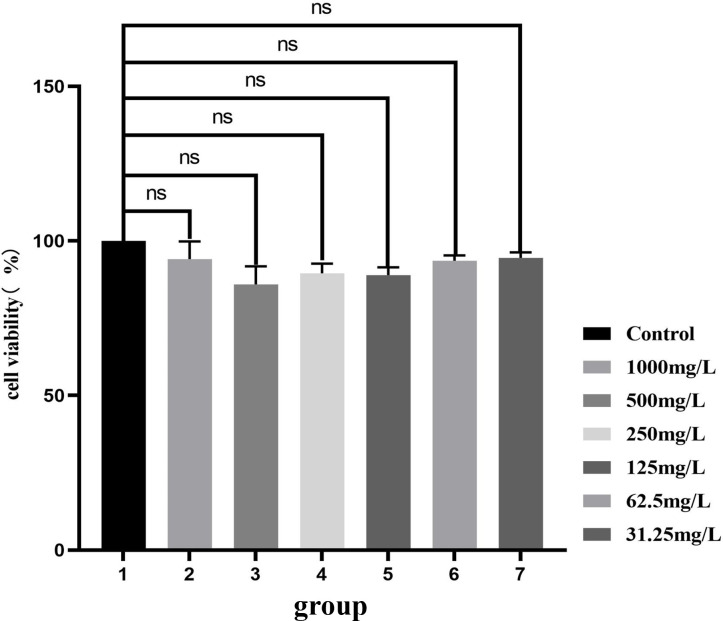
Cytotoxicity test.

In Vitro Cytotoxicity Tests (MTT) method was used to evaluate the potential cytotoxicity of the prepared material sample on mouse fibroblast 3T3 cells. As shown in Fig. 9, MTT cytotoxicity assays were performed using materials at different concentrations. Analysis revealed there is no significant difference in cell survival rates between the experimental group and control group (*P* > 0.05) (31). Consequently, it can be inferred that the synthesized photocatalytic materials exhibit no deleterious effects on normal cells and are devoid of toxicity. Hence, they pose no harm to animals during antibacterial processes.

## DISCUSSION

The aforementioned experimental data provide compelling evidence for the successful synthesis of Bi_12_ZnO_20_ material. The material’s photocatalytic antibacterial performance has been demonstrated to be superior through multiple laboratory experiments, and through the use of natural light. The material demonstrates remarkable photocatalytic antibacterial efficacy when tested on real wastewater samples. The generated free radicals in the reaction are sequentially eliminated, which unequivocally demonstrates free radicals indispensable role in the antibacterial response. The MTT experiment demonstrated the absence of significant cytotoxicity. The antibacterial experiment involving live organisms could not be conducted due to the laboratory conditions. In the future, there will be an increased opportunity for experimenting with highly pathogenic bacteria, such as *Mycobacterium tuberculosis*, and for exploring more practical scenarios, such as intensive care units and other medical environments.

### Conclusions

The synthesis method of nanostructured materials is facile and demonstrates exceptional photocatalytic performance as well as remarkable antibacterial efficacy. The bacteria can be rapidly and efficiently eradicated, whereas the survival rate of fungi can also be effectively controlled. Moreover, Bi_12_ZnO_20_ negligible cytotoxicity toward cells. Additionally, the photocatalytic antibacterial approach effectively addresses fungal antimicrobial resistance. Furthermore, the insolubility of this material in water enables efficient recycling to prevent secondary pollution and facilitates effective fungal control. Overall, these findings present promising prospects for development in both environmental and antibacterial domains.

## Data Availability

Data will be made available on request.

## AFTER EPUB

*[This article was published on 9 July 2024 with an error in the ORCID numbers. Shuai Liu's ORCID was corrected in the current version, posted on 15 July 2024.]*
